# University of Pennsylvania

**Published:** 1843-02-04

**Authors:** Henry Sargeant


### CLINICAL LECTURES AND REPORTS.

### UNIVERSITY OF PENNSYLVANIA.

### CLINIC OF DR. W. POYNTELL JOHNSTON.

*At the Locust Street Dispensary, Jan.* 14, 1843.

[Reported from notes by Henry Sargeant.]

LECTURE I.

(Concluded.)

Case IV.—*Lacrymal tumour—Operation—Occlusion of the nasal duct—Obliteration of the lower portion of the lacrymal sac—Great epiphora—Perforation of the os unguis—Cure.*—The patient now presented to you, gentlemen, Eliza Roberts, aged 25, of a lymphatic temperature, at the age of 15 was operated upon in England for a lacrymal tumour of the left eye. An incision was made into the lacrymal sac, and an effort made to pass a style into the nasal duct, this attempt proved unsuccessful and the disease was left to itself—the position of the cicatrix will show you how impossible it was for this operation to have succeeded. The incision was made too near the floor of the orbit. The introduction of a style into the nasal duct, through an opening in this situation would be impracticable. Since the operation the epiphora has greatly increased, but there has been no return of the lacrymal tumour. The eye was so constantly suffused with tears, that when she first presented herself at the clinic, five weeks since, you may recollect that she could neither read nor sew—her whole employment consisted in mopping the eye with her handkerchief. We endeavoured to introduce an instrument from the nose into the lacrymal sac, it passed as far as the floor of the orbit, and was there invincibly arrested. A probe was passed through each of the puncta lacrymalia, and through each reached the upper portion of the sac, where it was arrested. Water thrown by a syringe into the lower punctum, was ejected in a jet by the upper one. From these facts we concluded that the puncta and ducts, although smaller than usual, were not obliterated—that they opened into a remnant of the sac, although this remnant appeared to be smaller—and that it was necessary, in order to relieve the distressing infirmity under which the patient was suffering, either to re-establish the natural route of the tears, or to open a new channel of communication with the nose. We decided, therefore, to open the remains of the sac, if such could be found, and to endeavour to pass an instrument through the nasal duct; if this attempt failed, as we anticipated, the operation would be concluded by the formation of an artificial passage. We pointed out to you the various operations which have been suggested for this purpose, and decided in favour of a perforation of the os unguis.

The tendon of the orbicularis was rendered tense by drawing the muscle outwards—the tendon was carried slightly backwards by the flat surface of a straight bistourie, which was then plunged into the sac, and directed towards the nasal duct—the point did not penetrate into the duct—the bistouri was then withdrawn in such a way as to enlarge the incision in a direction parallel to the straight tendon.

Upon introducing a probe it was found, as was expected, that only the upper portion of the sac remained, into which the lacrymal canals opened; an ineffectual effort was again made to pass an instrument into the nasal duct. A plaque of horn, such as was employed by Mr. Hunter and Dr. Physick, in fact, the identical one used by Dr. Physick, was then introduced through the nostril, as a point d'appui, and the os unguis being fairly exposed, an effort was made to remove a circular piece from it by means of a punch. It was found, however, that owing to the projection of the process of the upper maxilla, the punch could not be made to act perpendicularly upon the bone. The plaque of horn was consequently removed, and the os unguis was perforated by a drill, shaped like a cobbler's awl and so managed as not to pierce the bone but to remove a circular piece by attrition. The wound was kept open for twenty-four hours by the introduction of charpie, and a style was then passed into the artificial opening in the bone; the epiphora was at once relieved, the patient was found reading on the third day, without inconvenience; the general health rapidly improved; the redness and puffiness of the lower lid, which caused so much deformity, disappeared; and she represents herself as entirely cured. We should be wrong, however, in offering this case as one of complete cure. She can read, sew, and attend to all the ordinary duties of life without the least inconvenience, and without any appearance of epiphora, but upon exposure to air, or to a strong light, or to the heat of the fire, the eye again becomes slightly suffused with tears. How shall we account for this? The tears pass freely into the nose from the remaining portion of the sac, the nostril is as moist as that on the opposite side, but we have already mentioned that the lacrymal puncta and ducts were themselves diminished in calibre. We will again examine their condition. The instrument employed is, as you perceive, not the probe of Anal, but an inflexible probe of steel, for which I am indebted to Professor Horner, which is fitted to a handle, similar to the handles generally adapted to eye instruments. An instrument of this kind possesses real advantages over the ordinary probe—the surgeon is not only certain of the position and direction of the point of his probe, (of which he cannot be with a delicate and very flexible instrument) but he is able to manipulate with such a handle with greater security—the objections which may be urged against the employment of an inflexible instrument from the injury it may inflict, attach of couse to the want of anatomical knowledge or want of skill of the operator, and not to the operation itself.

This small inflexible probe when introduced into the lower punctum passes readily to the sac, but at the moment of entering the sac it appears to encounter an obstacle. Each time that it is withdrawn and reintroduced we meet with the same difficulty; there is clearly at this point an obstruction, apparently a valvular fold of the mucous membrane. The introduction of the instrument is unattended by pain. In attempting to probe the upper duct, you perceive that this instrument is arrested at the punctum, which is much smaller than natural, we are compelled to employ the finest probe of Anal, which you perceive passes into the sac without the slightest difficulty. We have here a contraction of both the canals by which the tears are conveyed to the sac. Between the sac and nostril, a free communication has been established by an operation; this is shown by the fact that the left nostril is as moist as the right, and that the epiphora, from which she suffered so much, has entirely disappeared. The lacrymal ducts then have a sufficient calibre to convey to the sac the ordinary amount of tears secreted during the repose of the organ, and from the sac these tears pass freely into the nose, but when owing to any excitement the amount of tears becomes greatly augmented, the whole of the fluid cannot be transmitted through the contracted duct to the sac, and the excess exhibits itself as epiphora.

The cure in this case then is not complete—it is not sufficient to open a communication between the sac and the nostril, we must see that the channels by which the tears are conveyed to the sac are free also, and possess their natural calibre. The treatment will be continued by the introduction of probes, to dilate these canals, augmenting gradually the size of the probe, until the dilatation is complete.

Case V.—*Syphilis three years since—Great exposure during a whaling voyage—Caries of the upper maxilla.*—This young man contracted a chancre just prior to a whaling voyage, for which he shipped three years since. During the voyage he was treated by his shipmates, and took various preparations containing mercury, which were found in the medicine chest. The chancre became indurated, and finally healed, leaving an induration behind it. During the voyage he was much exposed in open boats, and, upon the return passage, suffered greatly from a swelled face. Upon his arrival he presented himself at the clinic. His appearance was then cachectic; his strength prostrated; his appetite gone, and digestion impaired. The face was enormously swollen; the mouth could with difficulty be opened; the fetor of the breath was extreme; the gums, in a spongy condition, bled profusely on the slightest touch; a fistula existed at the internal canthus of the left eye, from which mucus and tears could be forced. A probe introduced into this, encountered a small scale of denuded bone. On forcing the finger into the mouth, a large piece of dead bone could be felt projecting into the mouth; this was removed, and proved to be a portion of the tuberosity of the maxilla, about an inch in length, in a carious condition. The hæmorrhage which followed the removal of this fragment was alarming from its quantity; it could only be arrested by tamponing the cavity from which it was removed, with charpie impregnated with the muriated tincture of iron. The patient was at once placed upon the iodide of potassium, of which he took twenty grains daily in a half-pint of hop-tea, for two weeks. At the end of this time, a great improvement had taken place: the fistula lacrymalis had healed entirely; the swelling of the face had disappeared; the colour, appetite and strength had returned; the sponginess of the gums had diminished; in a word, all the symptoms had been ameliorated. He was directed to continue the iodide of potassium, and he now informs us that his health since that period has been entirely maintained. He presents himself to-day, complaining of a fœtid discharge from the left nostril. On introducing an instrument we encounter a fragment of denuded bone; you can hear the click of the instrument against it. You perceive that the various forceps we employ slip off from it; it does not project sufficiently to be grasped. We have at length seized it with a small pair of Gibson's bullet-forceps. Upon making traction, a depression is produced at the internal canthus of the eye; the tears flow over the cheek, and the patient complains of great pain in the infra orbital nerve. It is too firmly attached to be drawn away without great violence.

This is clearly a case of caries of the upper jaw, following *syphilis*—a syphilis which has become *constitutional*, as exhibited by the persisting induration, &c.—a syphilis which, in its primary form, was treated by a free, if not injudicious, use of *mercury*.

Time will not permit us, on this occasion, to examine some very interesting questions connected with this case. We can merely allude to them: Is this disease of the bone a simple consequence of chancre? Is it simply caused by the mercury which was administered? or is it the result of these two influences conjoined? To these questions we cannot, for the moment, enter upon a reply. Similar affections of the bones are, however, so frequently presented here, that we can scarcely fail of another opportunity of studying this subject, which is practically of great importance. The remaining carious fragments appear to consist of the nasal process of the upper maxilla, with a portion of the orbital plate, and to involve the cavity for the lodgement of the lacrymal sac, as well as the infra-orbital canal. Great violence would be required to detach it at present, and this violence might cause some injury to the lacrymal sac and infra-orbital nerve. It will be better, therefore, to permit nature to separate the diseased bone more thoroughly before attempting its removal. As the health of the patient is improving daily, nothing will be lost by the delay of a week or two. The same general treatment, under which he at first improved so rapidly, will be resumed.

℞. Potass. Iodidi, ℈iv,

In. Chart. No. iv. Die.

℞. Humuli Lupuli, ℥ij.

In. Chart. No. iv. Die.

S. Make half a pint of hop-tea with each paper of hops; strain the tea; add and dissolve in it one of the powders of iodide of potassium, and administer a wine-glassfull every 6 hours. Repeat this during the three succeeding days.

Case VI.—*Extraction of second large molar tooth of lower jaw followed by great swelling, and necrosis of the alveolar processes—Abscess near the chin—through the opening, the ramus of the jaw can be felt denuded.* —Elizabeth Barker, æt. 26, of lymphatic temperament, and great nervous irritability; otherwise in the enjoyment of good general health, had the second molar tooth of the left side of the lower jaw extracted about three months since. The extraction was followed by violent pain, which has continued, although less in degree, ever since. In the course of a week, a large swelling was perceived opposite the point from which the tooth was extracted. This swelling, at first entirely hard, gradually advanced towards the chin, and become more soft; finally a red fluctuating spot appeared beneath the ramus of the jaw, opposite the cuspid tooth of the lower side. This was opened by her physician, Dr. Page, with great relief to the patient. The swelling still occupies, however, the whole ramus of the jaw, and is sensible upon pressure. But the sensibility, is not so great as you might suppose from the shrinking of the patient. She is nervous to an extreme—nervous, not only because of the existence and duration of a real pain, but because the swelling prevents her from being properly nourished, and because the pain has been alleviated, prior to the formation of an abscess, by the continual administration of laudanum. These combined causes have produced a nervous prostration, which renders the patient remarkably timid and apprehensive. The mouth can only be opened to a moderate extent; the breath is extremely fetid; the finger introduced feels distinctly on the inside of the cavity, from which the tooth was removed, an exposed mass of bone, which is moveable. You perceive that it is easily extracted with a pair of tooth forceps, now that the patient has summoned up sufficient courage to open her mouth. The portion of bone removed, in length about an inch and a half, consists of the inner margin of apparently three alveolar processes. When we introduce a probe into the opening of the abscess, near the chin, it encounters at once a denuded bone, and can be directed backwards along this denuded bone, on the inner side of the ramus of the jaw, until its point can be felt by the finger, introduced into the mouth, opposite to the gap left by the removal of the dead alveolar process. It is arrested, however, by the mylohyoid muscle, and does not penetrate the mouth.

This denuded portion of bone is not moveable; the line of the teeth remains unbroken. Consequently, it cannot be regarded as a fragment detached from the bone and necrosed, as was the case with the fragment which we have removed. We must consider it as a mere exposed surface of bone—exposed by an abscess—which abscess, originating in an injury to the bone itself, has fused down in contact with the bone. A slight exfoliation may occur from this, after which granulations will appear and organize, and the cure will probably be completed without further recourse to instruments.

This case contrasts completely with the last presented. In the one, a general cause produced a morbid action in the upper jaw, terminating in caries; in the other, a local injury detached a fragment of the lower jaw, which in consequence was deprived of vitality, died, was necrosed; while an abscess, in contact with a remaining portion of the bone, separated the periosteum, cut off from the surface the proper supply of blood through the periosteal vessels, and also terminated in necrosis—the necrosis of merely the surface of the ramus of the jaw.

